# Development and validation of nomograms for predicting survival in patients with de novo metastatic triple-negative breast cancer

**DOI:** 10.1038/s41598-022-18727-2

**Published:** 2022-08-29

**Authors:** Mao-Shan Chen, Peng-Cheng Liu, Jin-Zhi Yi, Li Xu, Tao He, Hao Wu, Ji-Qiao Yang, Qing Lv

**Affiliations:** 1 Department of Breast Surgery, West China Hospital of Sichuan University, 37 Guoxue Street, Chengdu, 610041 People’s Republic of China; 2 Department of Breast Surgery and Thyroid Surgery, Affiliated Suining Central Hospital of Chongqing University, 127 Desheng Road West, Suining, 629000 People’s Republic of China

**Keywords:** Breast cancer, Breast cancer, Cancer therapy

## Abstract

Metastatic triple-negative breast cancer (mTNBC) is a heterogeneous disease with a poor prognosis. Individualized survival prediction tool is useful for this population. We constructed the predicted nomograms for breast cancer-specific survival (BCSS) and overall survival (OS) using the data identified from the Surveillance, Epidemiology, and End Results database. The Concordance index (C-index), the area under the time-dependent receiver operating characteristic curve (AUC) and the calibration curves were used for the discrimination and calibration of the nomograms in the training and validation cohorts, respectively. 1962 mTNBC patients with a median follow-up was 13 months (interquartile range, 6–22 months), 1639 (83.54%) cases died of any cause, and 1469 (74.87%) died of breast cancer. Nine and ten independent prognostic factors for BCSS and OS were identified and integrated to construct the nomograms, respectively. The C-indexes of the nomogram for BCSS and OS were 0.694 (95% CI 0.676–0.712) and 0.699 (95% CI 0.679–0.715) in the training cohort, and 0.699 (95% CI 0.686–0.712) and 0.697 (95% CI 0.679–0.715) in the validation cohort, respectively. The AUC values of the nomograms to predict 1-, 2-, and 3-year BCSS and OS indicated good specificity and sensitivity in internal and external validation. The calibration curves showed a favorable consistency between the actual and the predicted survival in the training and validation cohorts. These nomograms based on clinicopathological factors and treatment could reliably predict the survival of mTNBC patient. This may be a useful tool for individualized healthcare decision-making.

## Introduction

Breast cancer is the most common cancer in women and the leading cause of cancer-related deaths worldwide^1,2^. Among all newly diagnosed breast cancers, about 5–10% are de novo metastatic diseases^3^. The transition from phenotypic research to the exploration of intrinsic molecular subtypes has made a substantial transformation in the management of breast cancer^1^. Molecular-targeted therapies and precision medicine has greatly improved the prognosis of patients with specific genetic backgrounds^2–4^. However, the overall prognosis of metastatic breast cancer is still poor and heterogeneous. Triple negative breast cancer (TNBC) is the subtype of breast cancer with the most aggressive biological behavior, and is associated with a poor prognosis. Chemotherapy is the primary established systemic treatment for TNBC patients^5,6^. With the improvement of treatment strategies, the mortality rate for patients with de novo metastatic TNBC (mTNBC) is decreasing. However, the survival of this patients remains unsatisfactory^7–11^. Accurately predicting the prognosis of these patients can help guide clinical decision-making. At present, the American Joint Committee on Cancer (AJCC) TNM staging system is one of the best-established tools to predict survival for breast cancer. However, once the patients are confined to those with metastatic diseases, its prognostic value becomes limited. Therefore, an effective and accurate prognostic prediction model is urgently desired for this population.

Nomogram has been widely used for prognostic estimation in oncology. With the ability to generate individual probabilities of clinical events by integrating diverse prognostic and determinant variables, nomograms meet the demands for integrated biological-clinical models, and promotes the development of personalized medicine^12,13^. Nomograms can provide rapid computation through user-friendly digital interfaces, and output results that are easy to understand^14,15^. However, the survival predicting nomogram for mTNBC patients is needed. Therefore, in this study, we identified clinicopathologic factors associated with the prognosis of mTNBC patients using population-based data, and developed a nomogram based on these prognostic factors for individualized survival prediction.

## Materials and methods

This study protocol was approved by the Clinical Research Ethics Committee of the Affiliated Suining Central Hospital of Chongqing Medical University (No. LLSLH20210029). Written informed consent was waived for this study as for all patients have given prior informed consent to being registered in SEER database. This study was conducted according to the type 2a of prediction model studies and the article in accordance with the TRIPOD Statement^16^.

### Patients selection

After acquiring the access, we extracted eligible cases from the Research Plus Database of the Surveillance, Epidemiology, and End Results (SEER) program (https://seer.cancer.gov/, released April 2021), which consists of 18 population-based cancer registries. Cases that met the following inclusion criteria were generated using SEER*Stat Version 8.3.9 software: female, diagnosed from 2010 to 2017, age at diagnosis was older and equal to 18-year-old, pathologically confirmed as breast carcinoma, breast cancer as the first primary unilateral tumor, and the AJCC stage IV. Inflammatory breast cancer was allowed to be included. Cases with data obtained from death certificates or autopsy reports, or those without follow-up information were excluded. Patients’ unknown of race, marital status, tumor stage, node stage, histology, or history of breast surgery were excluded.

### Variables

We extracted the demographic features (including year of diagnosis, age at diagnosis, race, and marital status), clinicopathological characteristics (including histological type, tumor stage, node stage, TNM stage, bone metastasis, lung metastasis, liver metastasis, brain metastasis, and breast cancer subtype), treatment (including breast surgery, radiotherapy, and chemotherapy), and survival data (including survival months, vital status, etc.) of each case. Patients were grouped into five groups according to the age of diagnosis: 18–40 years old, 41–50 years old, 51–60 years old, 61–60 years old, and > 70 years old. Patients were classified as invasive ductal carcinoma (IDC, Code: 8500/3) and invasive lobular carcinoma (ILC, Code: 8522/3)/Others according to the International Classification of Diseases for Oncology third edition (ICD-O-3). The tumor TNM stage classification was based on the AJCC breast cancer system 7th edition.

The main outcomes of this study were breast cancer-specific survival (BCSS) and overall survival (OS). BCSS was defined as the interval (month) from the diagnosis to the breast cancer-related death, with lose of follow-up or death of other causes as censored data. OS was defined as the interval (month) from the diagnosis to death of any cause, with lose of follow-up was as censored data.

### Statistical analysis

Patients were randomly divided into the training and the validation cohorts at the ratio of 7:3. Chi-square test was used to determine the consistency of clinicopathological characteristics between the training and the validation cohorts. Parameters with a *P* value less than 0.1 in univariate Cox analysis or with a clinical consideration of potential prognostic factors were included in the multivariable Cox model to identify independent prognostic factors in the training cohort. The nomograms to predict 1-, 2-, and 3-year BCSS and OS were constructed based on the independent prognostic factors. The performance of the nomograms was evaluated in the training set and the validation set, respectively. The concordance index (C-index), time-dependent receiver operating characteristic (ROC) curve, and the area under the ROC curve were used to evaluate the distinguishing ability of the nomograms. The C-index and AUC value range from 0 to 1, and a higher value indicates a stronger predictive ability, and the value between 0.7 and 0.9 is generally considered to have well identification ability. The calibration curves were used to evaluate the accuracy of point estimates of nomogram-predicted survival with the actual survival. Bootstrap resample method (B = 1000) was used for calibration curve plot. The Survival curves were plotted by Kaplan–Meier method. Statistical analyses and figure plots were conducted by R software version 4.0.3 (www.r-project.org) using the packages of ‘survminer’, ‘survival’, ‘rms’, and ‘riskRegression’. All statistical analyses were two-sided, and a *P* value of less than 0.05 was considered statistically significant.

### Ethics statement

This study protocol was approved by the Clinical Research Ethics Committee of the Affiliated Suining Central Hospital of Chongqing Medical University (No. LLSLH20210029).

## Results

### Patient characteristics

**Figure 1:**
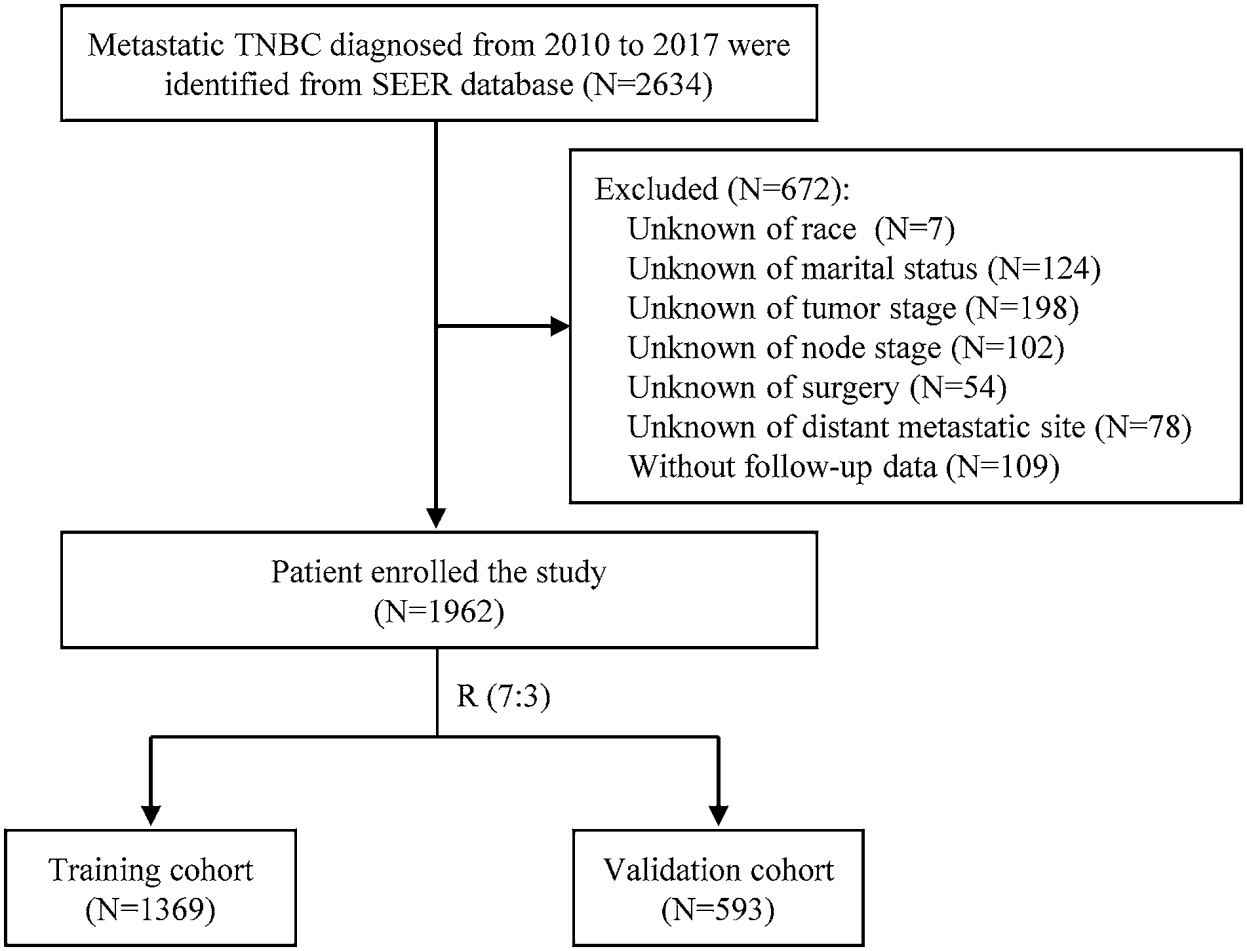
Flow chart of patient selection.

A total of 1962 patients met the criterial and were included in our analyses (Fig. 1). The demographic characteristics, clinicopathological features, and treatments of all patients were summarized in Table 1. Among all patients, the median age at diagnosis was 59 years (IQR: 50–69 years). Most (67.79%) of the patients were white. The percentage of distance metastasis of bone, lung, liver, and brain were 44.14%, 39.40%, 27.12%, and 9.93%, respectively. 864 (44.04%) patients received primary breast surgery including mastectomy (628 patients, 32.01%) and breast-conserving surgery (236 patients, 12.03%). 703 (35.83%) patients received radiotherapy, and 81.14% (1592) of patients received chemotherapy. Patients were randomly allocated into the training cohort (N = 1369) and the validation cohort (N = 593), and the distributions of clinicopathological features between the two cohorts were balanced (Table 1).

**Table 1:** Clinicopathological characteristics of metastatic TNBC in the training and validation cohorts.

Characteristics	Total cohort N = 1962	Training cohort N = 1369	Validation cohort N = 593	P value
**Year of diagnosis**				
2010–2013	959 (48.88)	666 (48.65)	293 (49.41)	0.757
2014–2017	1003 (51.12)	703 (51.35)	300 (50.59)	
**Age at diagnosis (years)**				
18–40	196 (9.99)	135 (9.86)	61 (10.29)	0.939
41–50	306 (15.60)	217 (15.85)	89 (15.01)	
51–60	542 (27.62)	375 (27.39)	167 (28.16)	
61–70	467 (23.80)	322 (23.52)	145 (24.45)	
> 70	451 (22.99)	320 (23.37)	131 (22.09)	
**Race**				
White	1330 (67.79)	928 (67.79)	402 (67.79)	0.999
Black	632 (32.21)	441 (32.21)	191 (32.21)	
**Marital status**				
Married	881 (44.90)	626 (45.73)	255 (43.00)	0.265
Unmarried	1081 (55.10)	743 (54.27)	338 (57.00)	
**Histology**				
IDC	1549 (78.95)	1079 (78.82)	470 ((79.26)	0.826
ILC/Others	413 (21.05)	290 (21.18)	123 (20.74)	
**Tumor stage (AJCC 7th)**				
T1	198 (10.09)	134 (9.79)	64 (10.79)	0.528
T2	582 (29.66)	412 (30.09)	170 (28.67)	
T3	372 (18.96)	250 (18.26)	122 (20.57)	
T4	810 (41.28)	573 (41.86)	237 (39.97)	
**Node stage (AJCC 7th)**				
N0	381 (19.42)	268 (19.58)	113 (19.06)	0.852
N1	861 (43.88)	605 ((44.19)	256 ((43.17)	
N2	233 (11.88)	164 (11.98)	69 (11.64)	
N3	487 (24.82)	332 (24.25)	155 (26.14)	
**Bone metastasis**				
No	1096 (55.86)	775 (56.61)	321 (54.13)	0.310
Yes	866 (44.14)	594 (43.39)	272 (45.87)	
**Lung metastasis**				
No	1189 (60.60)	825 (60.26)	364 (61.38)	0.641
Yes	773 (39.40)	544 (39.74)	229 (38.62)	
**Liver metastasis**				
No	1430 (72.88)	996 (72.75)	434 (73.19)	0.843
Yes	532 (27.12)	373 (27.25)	159 (26.81)	
**Brain metastasis**				
No	1765 (89.96)	1233 (90.07)	532 (89.71)	0.811
Yes	197 (9.93)	136 (9.93)	61 (10.29)	
**No. of metastatic site**				
1	1383 (70.49)	962 (70.27)	421 (70.99)	0.803
2	420 (21.41)	295 (21.55)	125 (21.08)	
3	129 (6.57)	93 (6.79)	36 (6.07)	
4	30 (1.53)	19 (1.39)	11 (1.85)	
**Breast surgery**				
No	1098 (55.96)	764 (55.81)	334 (56.32)	0.832
Yes	864 (44.04)	605 (44.19)	259 (43.68)	
**Radiotherapy**				
No	1259 (64.17)	884 (64.57)	375 (63.24)	0.571
Yes	703 (35.83)	485 (35.43)	218 (36.76)	
**Chemotherapy**				
No	370 (18.86)	253 (18.48)	117 (19.73)	0.516
Yes	1592 (81.14)	1116 (81.52)	476 (80.27)	

*IDC* infiltrating ductal carcinoma, *ILC* infiltrating lobular carcinoma, *AJCC* American Joint Committee on Cancer.

### Identification of predictors in training set

**Figure 2:**
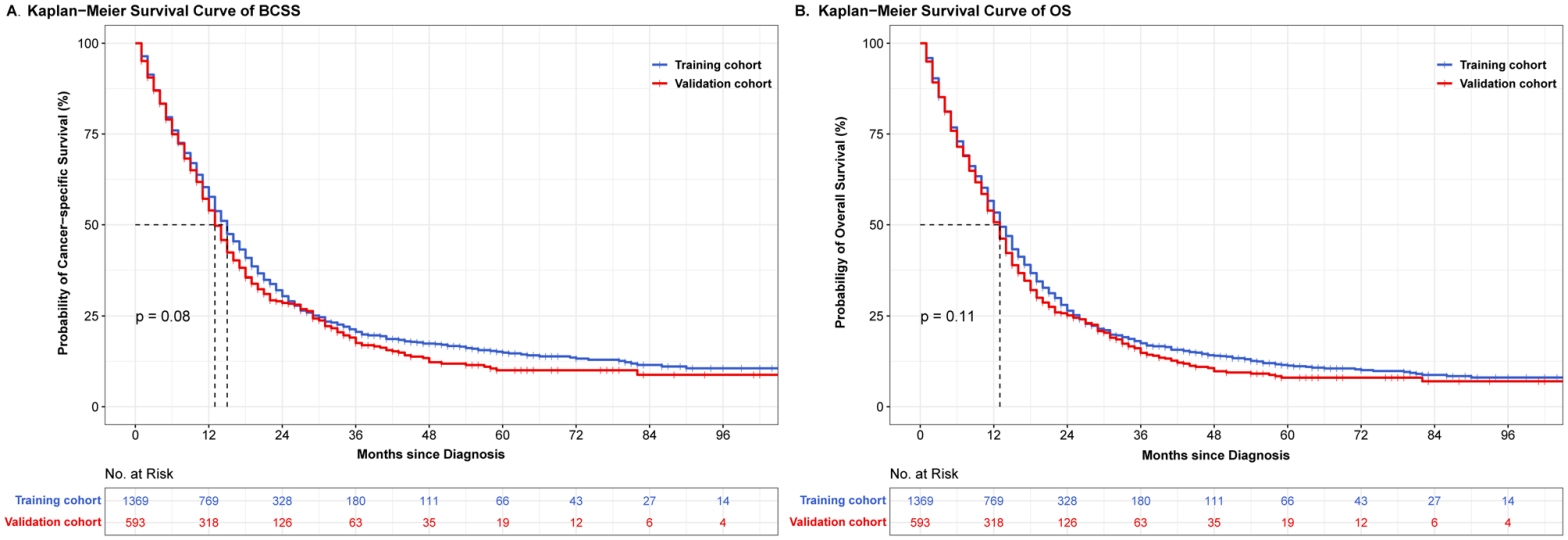
Survival curves of BCSS (**A**) and OS (**B**) in training and validation cohorts.

The median follow-up was 13 months (IQR: 6–22 months) for all patients. Among them, 1639 (83.54%) cases died of any cause, and 1469 (74.87%) cases died of breast cancer. There was no significant difference detected in the estimated 1-, 2-, and 3-year BCSS and OS between the total cohort, the training cohort, and the validation cohort (Table 2 and Fig. 2). The BCSS and OS rates between patients with different number of metastatic organs in training cohort were significantly different (Supplementary Fig. 1A,B and Table 1).

**Table 2:** The estimated 1-, 2-, and 3-year BCSS and OS in total, training, and validation cohort.

Outcomes	Total cohort	Training cohort	Validation cohort
**BCSS**			
1-year	56.55% (54.27–58.76%)	57.69% (54.97–60.31%)	53.93% (49.74–57.93%)
2-year	29.86% (27.69–32.05%)	30.42% (27.81–33.07%)	28.59% (24.75–32.54%)
3-year	19.71% (17.73–21.77%)	20.62% (18.23–23.12%)	17.55% (14.12–21.29%)
**OS**			
1-year	52.55% (50.32–54.74%)	53.37% (50.68–55.97%)	50.69% (46.59–54.63%)
2-year	26.03% (24.04–28.06%)	26.44% (24.05–28.88%)	25.14% (21.61–28.81%)
3-year	16.69% (14.94–18.52%)	17.47% (15.36–19.69%)	14.83% (11.84–18.15%)

*BCSS* breast cancer-specific survival, *OS* overall survival.

The results of univariate Cox analyses in the training cohort showed that age at diagnosis, marital status, tumor stage, node stage, bone metastasis, lung metastasis, liver metastasis, brain metastasis, breast surgery, radiotherapy, and chemotherapy were the potential prognostic factors for BCSS and OS (Table 3). Considering the interaction between the metastatic site and the number of metastatic organs, the metastatic organs were included in the Cox model to well investigate the impact of metastatic pattern on the survival. In multivariable Cox analysis, age at diagnosis, marital status, tumor stage, node stage, bone metastasis, liver metastasis, brain metastasis, breast surgery, and chemotherapy were the independent prognostic factors for BCSS and OS (Table 3). In addition, radiotherapy was significantly associated with the OS in patients with mTNBC (Table 3).

**Table 3:** Univariate and multivariable Cox analysis of BCSS and OS in training cohort.

Characteristics	Univariate analysis				Multivariable analysis			
	BCSS		OS		BCSS		OS	
	HR (95% CI)	*P* value	HR (95% CI)	*P* value	HR (95% CI)	*P* value	HR (95% CI)	*P* value
**Year of diagnosis**								
2010–2013	1 [reference]	–	1 [reference]	–				
2014–2017	0.94 (0.82–1.06)	0.274	0.93 (0.83–1.05)	0.239				
**Age (years)**								
18–40	1 [reference]	–	1 [reference]	–	1 [reference]	–	1 [reference]	
41–50	1.23 (0.96–1.59)	0.108	1.19 (0.93–1.52)	0.158	1.30 (1.00–1.68)	0.046	1.26 (0.99–1.61)	–
51–60	1.12 (0.88–1.42)	0.367	1.12 (0.90–1.40)	0.319	1.15 (0.90–1.46)	0.261	1.14 (0.91–1.43)	0.064
61–70	1.27 (0.99–1.61)	0.057	1.26 (1.00–1.59)	0.046	1.18 (0.92–1.51)	0.183	1.16 (0.92–1.45)	0.246
> 70	1.65 (1.30–2.10)	< 0.001	1.68 (1.34–2.11)	< 0.001	1.54 (1.21–1.98)	< 0.001	1.52 (1.20–1.92)	0.219
**Race**								
Black	1 [reference]	–	1 [reference]	–				
White	0.98 (0.85–1.11)	0.711	0.96 (0.85–1.09)	0.529				
**Marital status**								
Married	1 [reference]	–	1 [reference]	–	1 [reference]	–	1 [reference]	–
Unmarried	1.38 (1.22–1.56)	< 0.001	1.41 (1.25–1.59)	< 0.001	1.17 (1.03–1.33)	0.016	1.19 (1.05–1.34)	0.005
**Histology**								
IDC	1 [reference]	–	1 [reference]	–				
ILC/Others	1.10 (0.95–1.28)	0.218	1.11 (0.96–1.28)	0.152				
**Tumor stage**								
T1	1 [reference]	–	1 [reference]	–	1 [reference]	–	1 [reference]	–
T2	1.19 (0.94–1.51)	0.158	1.02 (0.82–1.27)	0.832	1.29 (1.01–1.64)	0.042	1.10 (0.89–1.38)	0.377
T3	1.35 (1.05–1.74)	0.020	1.15 (0.91–1.45)	0.243	1.30 (1.00–1.69)	0.050	1.11 (0.87–1.41)	0.387
T4	1.47 (1.17–1.85)	0.001	1.31 (1.06–1.61)	0.011	1.50 (1.18–1.92)	< 0.001	1.35 (1.08–1.64)	0.007
**Node stage**								
N0	1 [reference]	–	1 [reference]	–	1 [reference]	–	1 [reference]	–
N1	1.26 (1.05–1.50)	0.011	1.21 (1.02–1.42)	0.025	1.12 (0.93–1.34)	0.222	1.09 (0.92–1.29)	0.311
N2	1.26 (1.00–1.58)	0.052	1.18 (0.95–1.47)	0.127	1.27 (1.00–1.61)	0.047	1.22 (0.97–1.52)	0.083
N3	1.29 (1.06–1.56)	0.011	1.21 (1.01–1.45)	0.044	1.33 (1.08–1.63)	0.007	1.27 (1.04–1.54)	0.017
**Bone metastasis**								
No	1 [reference]	–	1 [reference]	–	1 [reference]	–	1 [reference]	–
Yes	1.30 (1.15–1.47)	< 0.001	1.30 (1.16–1.46)	< 0.001	1.27 (1.11–1.44)	< 0.001	1.27 (1.12–1.44)	< 0.001
**Lung metastasis**								
No	1 [reference]	–	1 [reference]	–	1 [reference]	–	1 [reference]	–
Yes	1.21 (1.07–1.37)	0.002	1.20 (1.07–1.35)	0.002	1.08 (0.95–1.23)	0.254	1.08 (0.95–1.22)	0.258
**Liver metastasis**								
No	1 [reference]	–	1 [reference]	–	1 [reference]	–	1 [reference]	–
Yes	1.61 (1.41–1.84)	< 0.001	1.59 (1.40–1.81)	< 0.001	1.64 (1.42–1.88)	< 0.001	1.63 (1.42–1.86)	< 0.001
**Brain metastasis**								
No	1 [reference]	–	1 [reference]	–	1 [reference]	–	1 [reference]	–
Yes	1.89 (1.57–2.29)	< 0.001	1.72 (1.43–2.07)	< 0.001	1.91 (1.56–2.34)	< 0.001	1.72 (1.41–2.10)	< 0.001
**Breast surgery**								
No	1 [reference]	–	1 [reference]	–	1 [reference]	–	1 [reference]	–
Yes	0.43 (0.40–0.52)	< 0.001	0.45 (0.40–0.51)	< 0.001	0.53 (0.46–0.61)	< 0.001	0.53 (0.47–0.61)	< 0.001
**Radiotherapy**								
No	1 [reference]	–	1 [reference]	–	1 [reference]	–	1 [reference]	–
Yes	0.78 (0.68–0.89)	< 0.001	0.76 (0.67–0.86)	< 0.001	0.87 (0.75–1.01)	0.063	0.87 (0.75–0.99)	0.039
**Chemotherapy**								
No	1 [reference]	–	1 [reference]	–	1 [reference]	–	1 [reference]	–
Yes	0.46 (0.39–0.53)	< 0.001	0.43 (0.37–0.50)	< 0.001	0.49 (0.41–0.58)	< 0.001	0.46 (0.39–0.54)	< 0.001

*BCSS* breast cancer-specific survival, *OS* overall survival, *HR* hazard ratio, *IDC* infiltrating ductal carcinoma, *ILC* infiltrating lobular carcinoma.

### Construction of the nomograms for BCSS and OS

**Figure 3:**
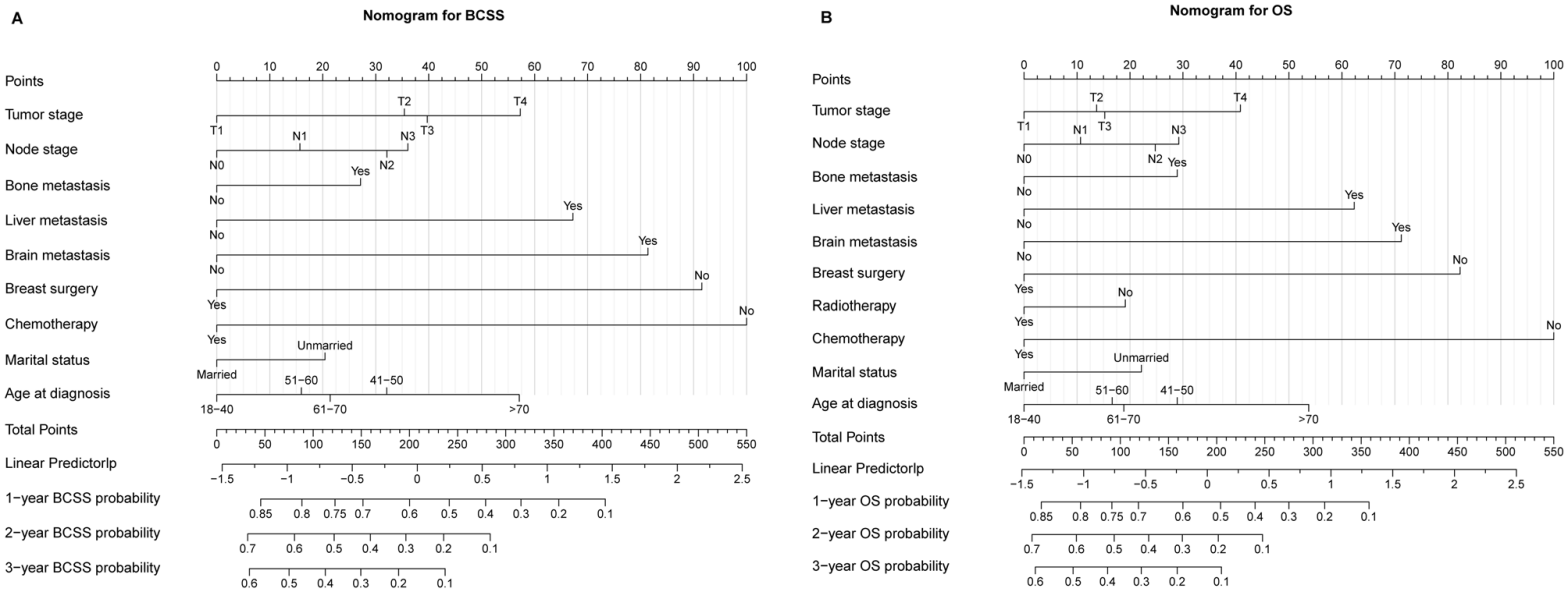
Nomograms for BCSS (**A**) and OS (**B**) in training cohort.

The nomograms were constructed based on the independent prognostic factors identified by the multivariable Cox model. Nine variables including tumor stage, node stage, bone metastasis, liver metastasis, brain metastasis, breast surgery, chemotherapy, marital status, and age at diagnosis were contained in the nomogram for BCSS (Fig. 3A). Ten variables including tumor stage, node stage, bone metastasis, liver metastasis, brain metastasis, breast surgery, radiotherapy, chemotherapy, marital status, and age at diagnosis were contained in the nomogram for OS (Fig. 3B).

### Validation of the nomograms

The C-indexes of the nomogram for BCSS and OS were 0.694 (95% CI 0.676–0.712) and 0.699 (95% CI 0.679–0.715) in the training cohort, and 0.699 (95% CI 0.686–0.712) and 0.697 (95% CI 0.679–0.715) in the validation cohort, respectively.

**Figure 4:**
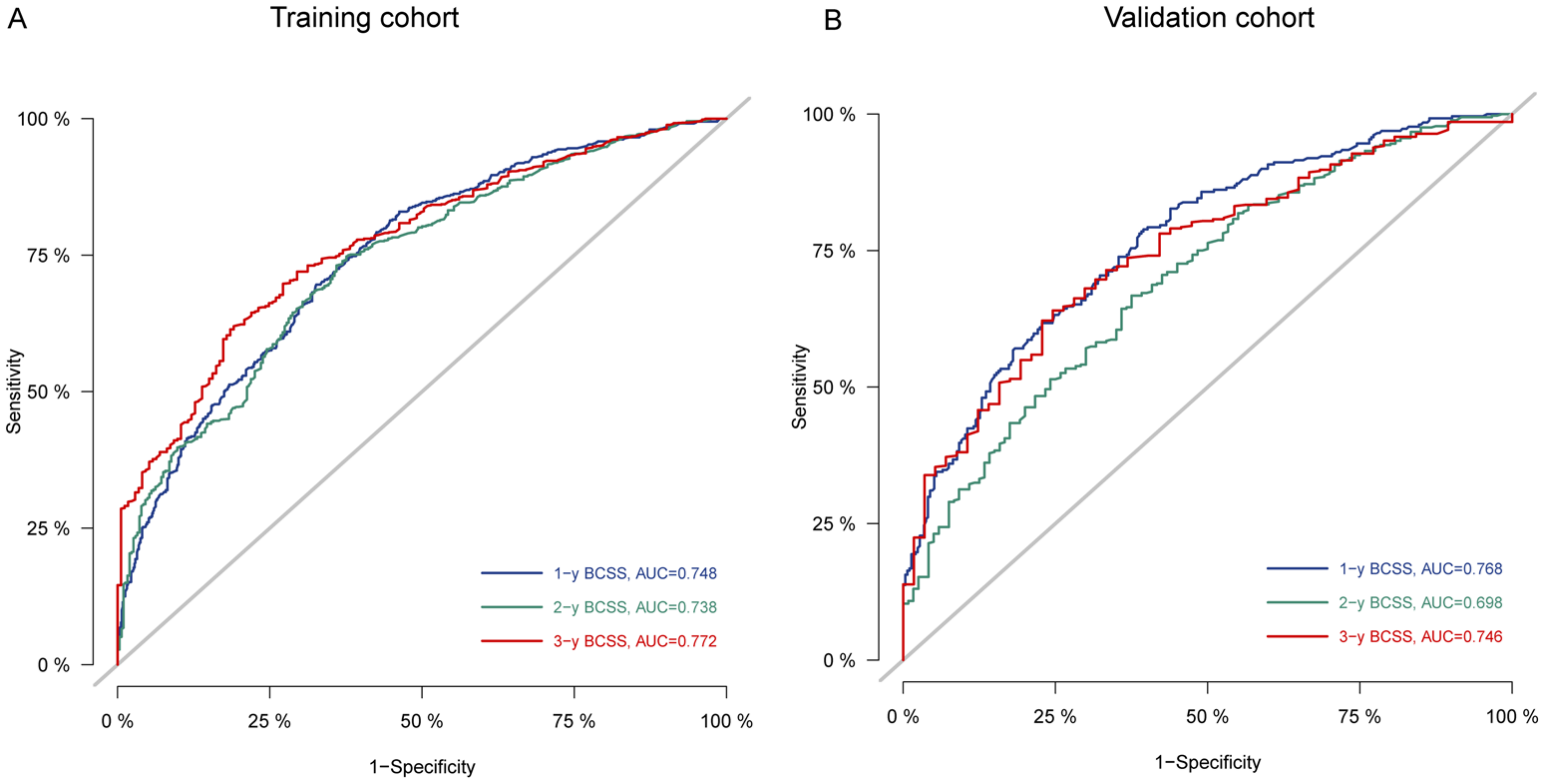
ROC curves and AUC for 1-, 2-, and 3-year BCSS in training (**A**) and validation (**B**) cohorts.

The time-dependent ROC was used to evaluate the point predictive values of the nomograms. In internal validation, the AUC values of the nomogram to predict 1-, 2-, and 3-year BCSS were 0.748 (95% CI 0.722–0.775), 0.738 (95% CI 0.706–0.769), and 0.772 (95% CI 0.738–0.806), respectively (Fig. 4A). In external validation, the AUC values of the nomogram to predict 1-, 2-, and 3-year BCSS were 0.768 (95% CI 0.730–0.807), 0.698 (95% CI 0.646–0.750), and 0.746 (95% CI 0.684–0.807), respectively (Fig. 4B). In the training cohort, the AUC values of the nomogram to predict 1-, 2-, and 3-year OS were 0.751 (95% CI 0.726–0.777), 0.747 (95% CI 0.716–0.777), and 0.783 (95% CI 0.749–0.817), respectively (Supplementary Fig. 2A). In the validation cohort, the AUC values of the nomogram to predict 1-, 2-, and 3-year OS were 0.768 (95% CI 0.731–0.806), 0.707 (95% CI 0.655–0.759), and 0.755 (95% CI 0.695–0.816), respectively (Supplementary Fig. 2B).

**Figure 5:**
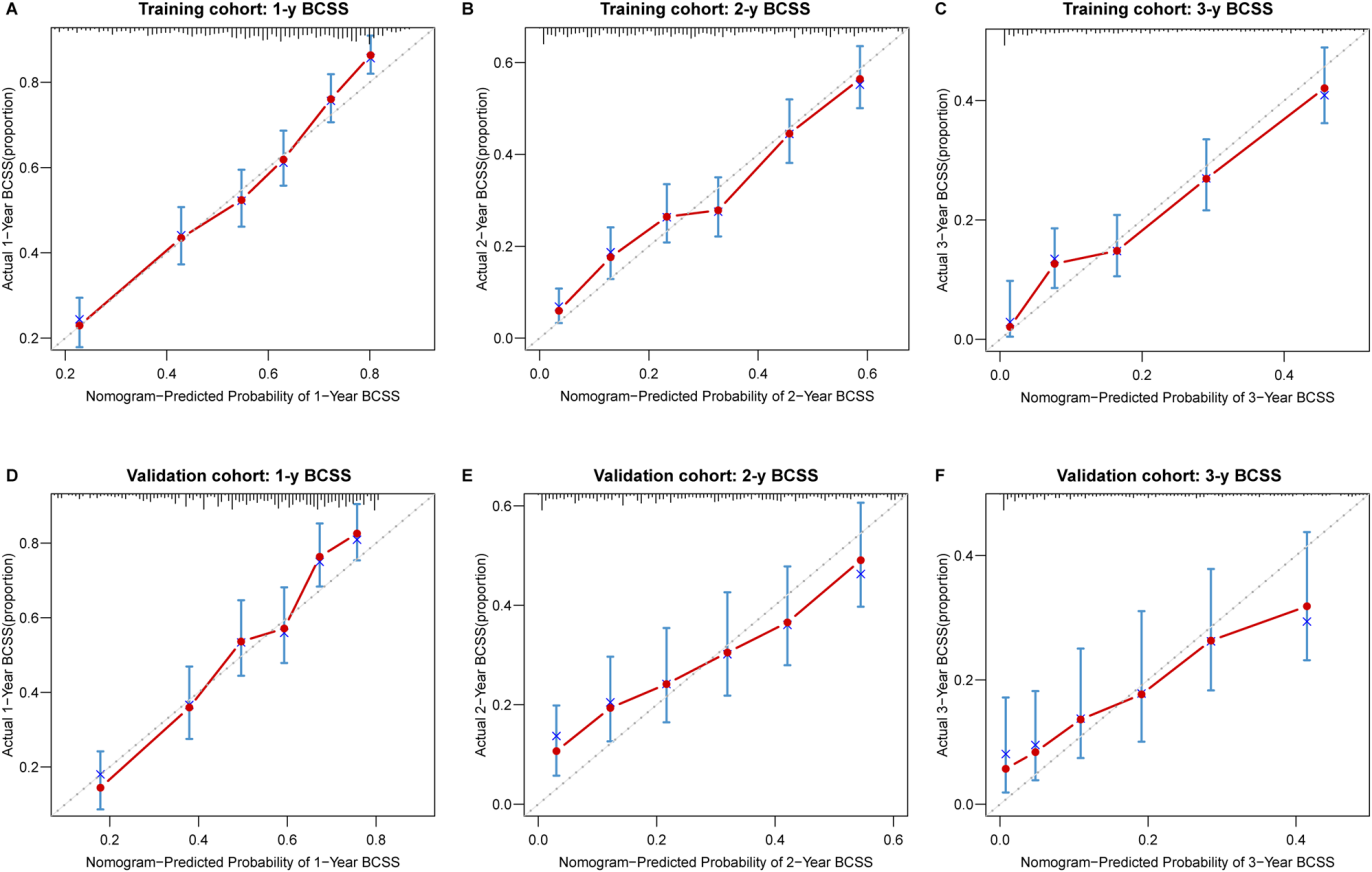
Calibration curves of 1-, 2-, and 3-year BCSS in training and validation cohorts. (**A**–**C**) Calibration curves of 1-, 2-, and 3-year BCSS in training cohort, respectively. (**D**–**F**) Calibration curves of 1-, 2-, and 3-year BCSS in validation cohort, respectively.

The 1-, 2-, and 3-year calibration curves of the nomogram for the prediction of BCSS demonstrated a good consistency in training cohort (Fig. 5A–C) and validation cohort (Fig. 5D–F). Similarly, the calibration curves of the nomogram for OS revealed a good consistency in two cohorts (Supplementary Fig. 3).

### Stratified survival analysis based on nomograms

**Figure 6:**
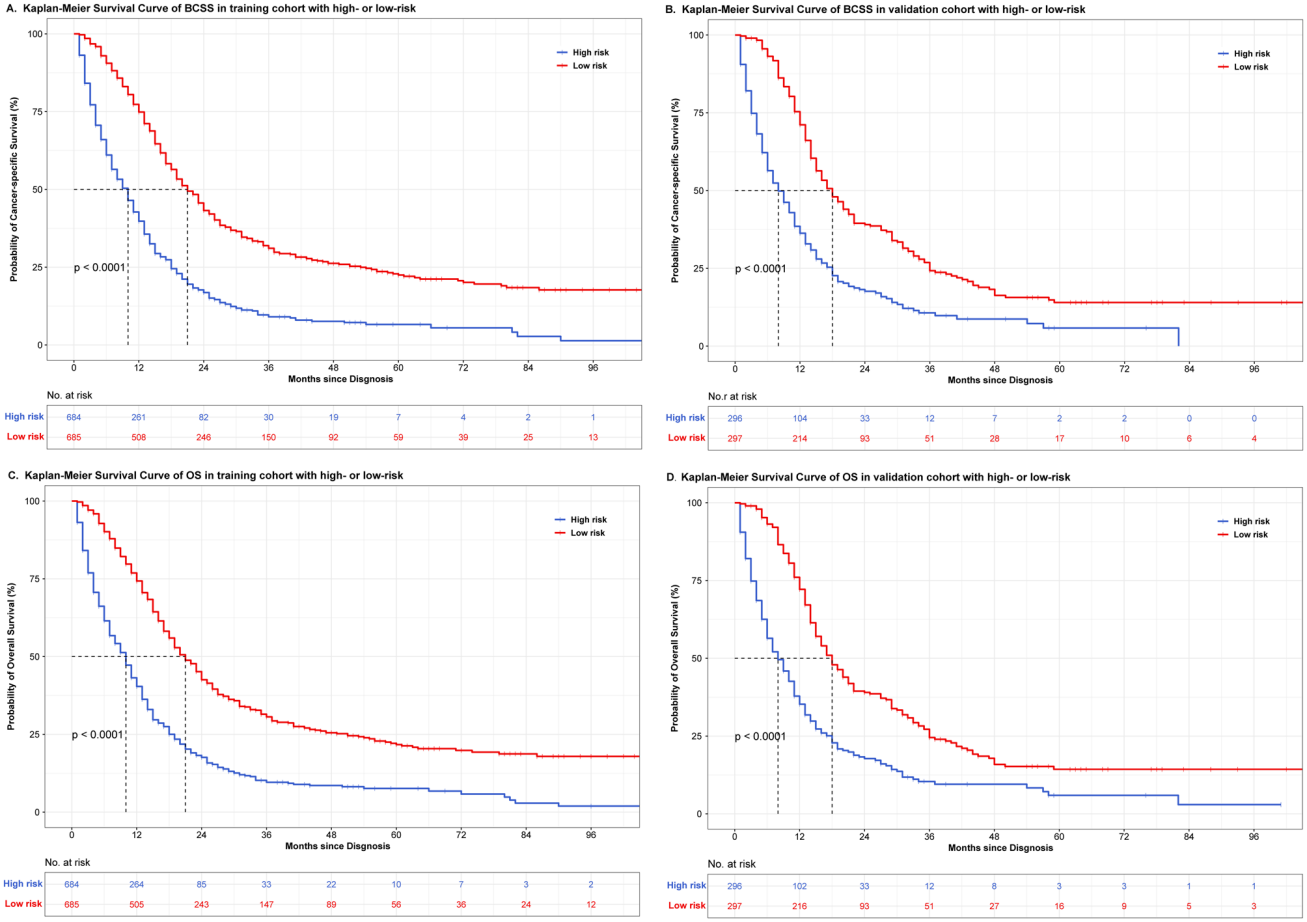
Survival curves of BCSS and OS stratified by nomogram estimated risk. (**A**, **B**) BCSS in training and validation cohort, respectively. (**C**, **D**) OS in training and validation cohort, respectively.

The risk score of each case in training and validation set were calculated based the nomograms for BCSS and OS. Patients were classified as low- and high-risk group with the cutoff of median risk score (BCSS: 110 points; OS: 90 points). The discrepancy of the median BCSS between low- and high-risk patients were 12 months and 9 months in training (21 months versus 9 months) and validation (17 months versus 8 months) sets, respectively (Fig. 6A,B). The discrepancy of the median OS between low- and high-risk patients were 11 months and 9 months in training (20 months versus 9 months) and validation (17 months versus 8 months) sets, respectively (Fig. 6C,D).

## Discussion

Metastatic breast cancer remains an incurable disease, although the survival has been improved in the past few decades thanks to advances in systemic treatment options^17^. The median overall survival for mTNBC is about 15 months^9^, and accurately estimating the prognosis of individual patients in this population can help medical care decision-making. We used data of the mTNBC patients extracted from the SEER database to identify the prognostic factors, and developed the nomograms to predict the 1-,2- and 3-year BCSS and OS. The nomogram showed good discrimination in both internal and external validations and is expected to provide favorable guidance for prognosis prediction and disease management. Previous studies have shown that a later time of diagnosis and treatment was associated with a better prognosis^18,19^. However, year of diagnosis was not associated with improved survival in the current multivariable Cox model, which might be related to the inherently poor prognosis of the disease and the insignificant improvement in treatment within a short period of time. Health gains and cost effectiveness are negatively related to age at diagnosis. Younger patients with stage IV breast cancer have better survival than their older counterparts^20,21^. In our analysis, we found that age was a significant prognostic factor for mTNBC patients.

Accumulated evidence has confirmed that race plays an independent prognostic role in TNBC patients, and that black women have a poorer survival than the white^22,23^. Comparing to white patients, black women had more advanced disease at diagnosis, had more germline BRCA mutations, had lower socioeconomic status, and received fewer treatments^24^. However, as indicated in our study, race was not an independent predictor of prognosis in mTNBC. Therefore, the racial/ethnic disparities in prognosis might be the result of unequal insurance coverage and access to care. Marital status is strongly associated with improved health and longevity. A growing body of evidence has shown that the mortality of unmarried breast cancer patients is higher than that of married patients, which may be explained married patients can get more mental and financial support from their partners^25,26^. Again, this conclusion was confirmed in our study. Although the needs of breast cancer patients can be partially provided by their children and relatives, not all of them can be provided. In unmarried patients, the marriage after the breast cancer diagnosis also has the positive impact on the survival^27^.

At present, the treatment strategy and prognostic prediction for invasive breast cancer patients are mainly based on the TNM staging system. According to our report, nodal stage does not affect the prognosis of mTNBC patients. Stage T4 breast cancers, including tumors with chest wall invasion (T4a), skin invasion consisting of ulceration or nodules (T4b) or both (T4c), and inflammatory breast cancer (IBC), had unfavorable influences on the prognosis. Besides, tumor histology were not independent predictors of prognosis in the multivariable analysis. In this cohort, the cumulative incidence of bone, liver, lung, and brain metastases were 41.13%, 39.78%, 26.97%, and 9.79%, respectively. Any site of distant metastasis except for lung confers a worse prognosis, and the survival was worse with the increased number of metastatic organs. Why is there no statistical significance in the effect of lung metastasis on survival in our data? TNBC is prone to visceral metastasis, which usually has more than two sites of metastasis simultaneously^5^. In this cohort data, over 52% of patients with lung metastasis had metastasis at other organs. The prognostic value of lung metastasis has changed for the interaction with other factors in the multivariable model, which explains why lung metastasis harmed survival in univariate analysis but not in multivariable analysis. Besides, there is also some discrepancy in the treatment sensitivity of different metastatic sites, which could change the prediction value of a variable^5,19^. In addition, number of lesions in a single metastatic organ may also affect patients’ outcome. This issue needs to be further studied.

Management of mTNBC is aimed at relieving symptoms and extending quality-adjusted life expectancy, and multidisciplinary collaboration is required. Generally, local treatments (surgery and radiation therapy) are not the mainstay of advanced breast cancer treatment, but can be very useful in certain situations. The survival benefits brought by resecting primary tumor in patients with metastatic breast cancer remains controversial, as suggested by some trials^28–32^. Radiation therapy has a crucial role in alleviating symptoms from bone, brain^33,34^, and should be prescribed in a multidisciplinary and individualized approach with dose and fractionation schedules depending on the severity of the lesions and the remaining life expectancy. Although previous researches and this analysis have indicated possibility improvement in survival contributed by radiation therapy^35^, the actual effect should be further validated. Despite less direct evidences about the prognostic value of radiotherapy on mTNBC patients, it should be considered for selected patients based on the pattern and metachronicity of the disease. In line with previous studies, our results suggested that chemotherapy promoted survival independently^36,37^. Chemotherapy has been the main treatment for TNBC, the change of chemotherapy regimens not only improve the prognosis, but also provide more treatment options. A phase III randomized clinical trial has investigated the efficacy and safety of cisplatin combined with nab-paclitaxel (AP) or gemcitabine (GP) as the first line treatment for metastatic TNBC, and the results demonstrated patients received AP had a longer PFS than that in patient treateated with GP regimen (9.8 months versus 7.4 months)^38^. Quite recently, while immunotherapy and targeted therapy has been emerging as novel treatment modalities for mTNBC^10,11,39^, further improvements in patients’ life expectancy and quality are foreseeable. KEYNOTE-355 trail has investigated the efficacy and safety of immunotherapy (pembrolizumab) added to chemotherapy in 847 advanced TNBC. In patients whose tumors expressed programmed death ligand (PD-L1), pembrolizumab could significantly longer survival than chemotherapy alone^40^. Besides, our previous study, have also shown that novel targeted therapeutic modalities may be an inspiring outlook in triple negative breast cancer^41^.

The value of local surgery in metastatic breast cancer is controversial. Several randomized clinical trials had investigated the efficacy of surgery in this population^28,29,42,43^. The results of these studies were inconsistent for the discrepancy in patient features, study design, and background between each study. But, the viewpoint of some patients who may benefit from surgery can be drawn in the modern era. Patients could be classified into a high- or low-risk group according to the nomograms, which could predict a relative worse or good outcome. The prediction tool considered several factors, which would avoid overemphasizing the value of surgery for mTNBC patients. Meanwhile, the prediction model could predict which patient received surgery had a relatively good outcome. Besides, some stage IV patients would accept surgery for local control, when presented with tumor growth, local infection, and bleeding.

In mTNBC patients, the ultimate aims of care are to optimize both quality and life span. The management of mTNBC is complex and, therefore, involvement of all appropriate specialties in a multidisciplinary team (including but not restricted to medical, radiation, surgical oncologists, imaging experts, pathologists, gynecologists, psycho-oncologists, social workers, nurses, and palliative care specialists), is crucial^44^.

## Limitations

Our study has several limitations. First, the SEER database does not provide details about chemotherapy and radiotherapy regimens, which may impact the survival or quality of life differently for mTNBC patients. Second, the information about metastatic involvement of specific organ sites is only collected at the time of initial presentation in SEER, and currently there is no longitudinal follow-up data to document subsequent organs affected. Third, SEER currently does not collect information on other sites of metastases such as distant lymph nodes, pleura, peritoneum, or skin. This information could assist in more specific prognostic assessment of the other metastatic groups. Fourth, the performance status (PS) of each patient were not provided in the SEER database, which was an important factor for clinical decision-making and survival. Finally, these nomograms were based on a retrospective set, and further validation in prospective clinical trials is needed.

## Conclusion

The nomograms have been established and validated for predicting BCSS and OS in TNBC patients with metastatic disease, which hold promises in realizing individualized prognostic prediction and identifying the high-risk patients who require more specialized treatment strategies and follow-up plans.

## Supplementary Information

Supplementary Information 1.

Supplementary Information 2.

Supplementary Information 3.

## Data Availability

The datasets analyzed during the current study are available from the SEER registry https://seer.cancer.gov/. Further inquiries of this study data can be directed to the corresponding author.

## Abbreviations

AJCC: American Joint Committee on Cancer
AUC: Area under the time-dependent receiver operating characteristic curve
BCSS: Breast cancer-specific survival
IBC: Inflammatory breast cancer
IDC: Invasive ductal carcinoma
ILC: Infiltrating lobular carcinoma
IQR: Interquartile range
mTNBC: Metastatic triple-negative breast cancer
OS: Overall survival
ROC: Receiver operating characteristic
TNBC: Triple-negative breast cancer
