# Supplementary figures and images for: Development and validation of nomograms for predicting survival in patients with de novo metastatic triple-negative breast cancer
Source: Sci Rep. 2022 Aug 29;12:14659. doi: 10.1038/s41598-022-18727-2 (PMC9424305; doi:10.1038/s41598-022-18727-2)

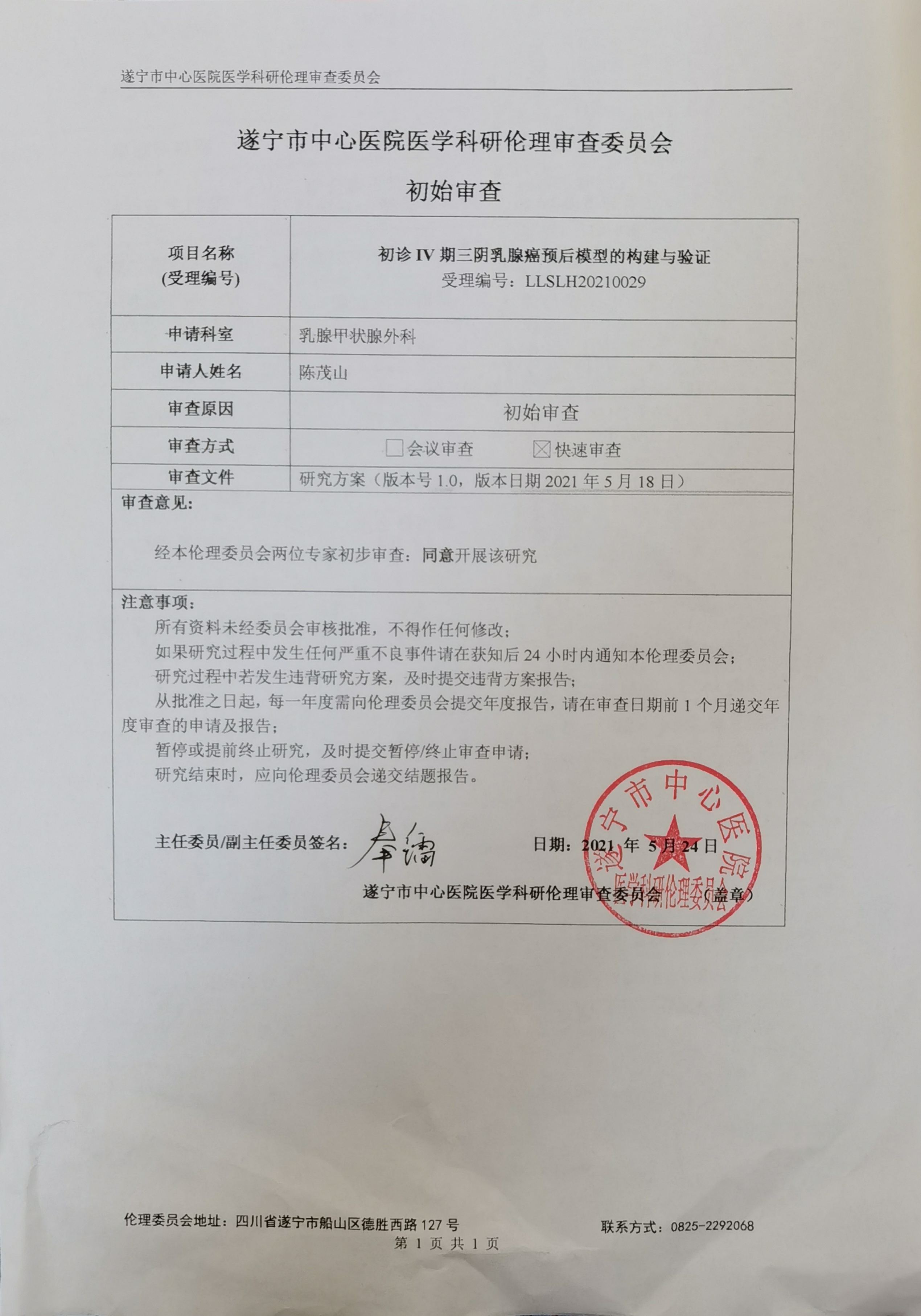

Supplement: Supplementary file 1 — Supplementary Information 1. [file 41598_2022_18727_MOESM1_ESM.jpg]
