# Supplementary material for: Development and validation of nomograms for predicting survival in patients with de novo metastatic triple-negative breast cancer
Source: Sci Rep. 2022 Aug 29;12:14659. doi: 10.1038/s41598-022-18727-2 (PMC9424305; doi:10.1038/s41598-022-18727-2)
Supplement: Supplementary file 3 — Supplementary Information 3. [file 41598_2022_18727_MOESM3_ESM.docx]

**Supplementary material**

**Supplementary table 1.** The estimated 1-, 2-, and 3-year BCSS and OS in patients with different metastatic organ number.

**Supplementary Fig 1.** Survival curves of BCSS and OS in training and validation cohort stratified by metastatic organ number.

**Supplementary Fig 2.** ROC curves and AUC for 1-, 2-, and 3-year OS in training and validation cohorts.

**Supplementary Fig 3.** Calibration curves of 1-, 2-, and 3-year OS in training and validation cohorts.

**Supplementary table 1. The estimated 1-, 2-, and 3-year BCSS and OS rates in patients with different metastatic organ number.**

| No. of metastatic organs | Training cohort | | |  | Validation cohort | | |
| --- | --- | --- | --- | --- | --- | --- | --- |
|  | 1-year | 2-year | 3-year |  | 1-year | 2-year | 3-year |
| One organ  BCSS  OS | 64.02%  59.78% | 36.49%  31.31% | 25.73%  22.18% |  | 64.46%  60.32% | 35.64%  31.38% | 22.65%  19.29% |
| Two organs  BCSS  OS | 46.28%  41.57% | 18.44%  15.89% | 10.00%  7.58% |  | 32.06%  31.20% | 13.45%  11.63% | 5.64%  4.43% |
| Three organs  BCSS  OS | 32.61%  29.03% | 9.11%  7.74% | 3.04%  2.58% |  | 21.18%  20.33% | NA  NA | NA  NA |
| Four organs  BCSS  OS | 26.32%  26.32% | 5.26%  5.26% | NA  NA |  | 0.00%  0.00% | NA  NA | NA  NA |

NA: not available.

**Supplementary Fig 1.** Survival curves of BCSS and OS in training and validation cohort stratified by metastatic organ number.


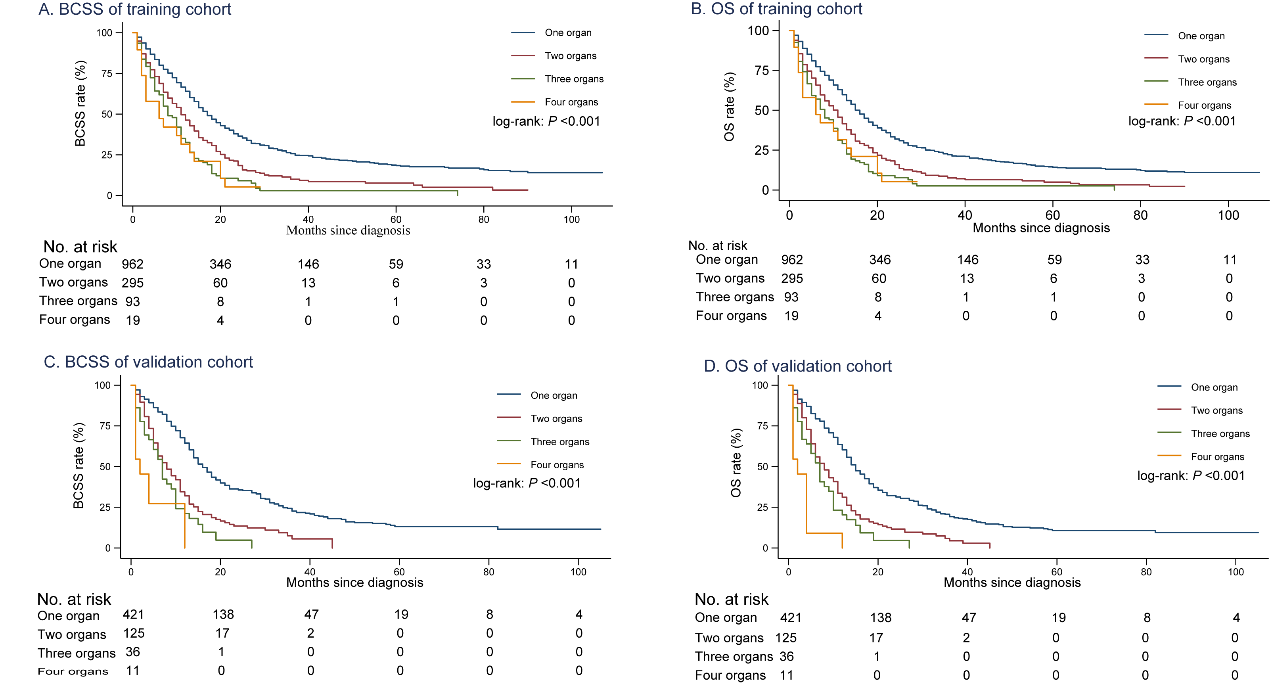


A. BCSS in training cohort; B. OS in training cohort; C. BCSS in validation cohort; D. OS in validation cohort.

**Supplementary Fig 2. ROC curves and AUC for 1-, 2-, and 3-year OS in training (A) and validation (B) cohorts.**

**
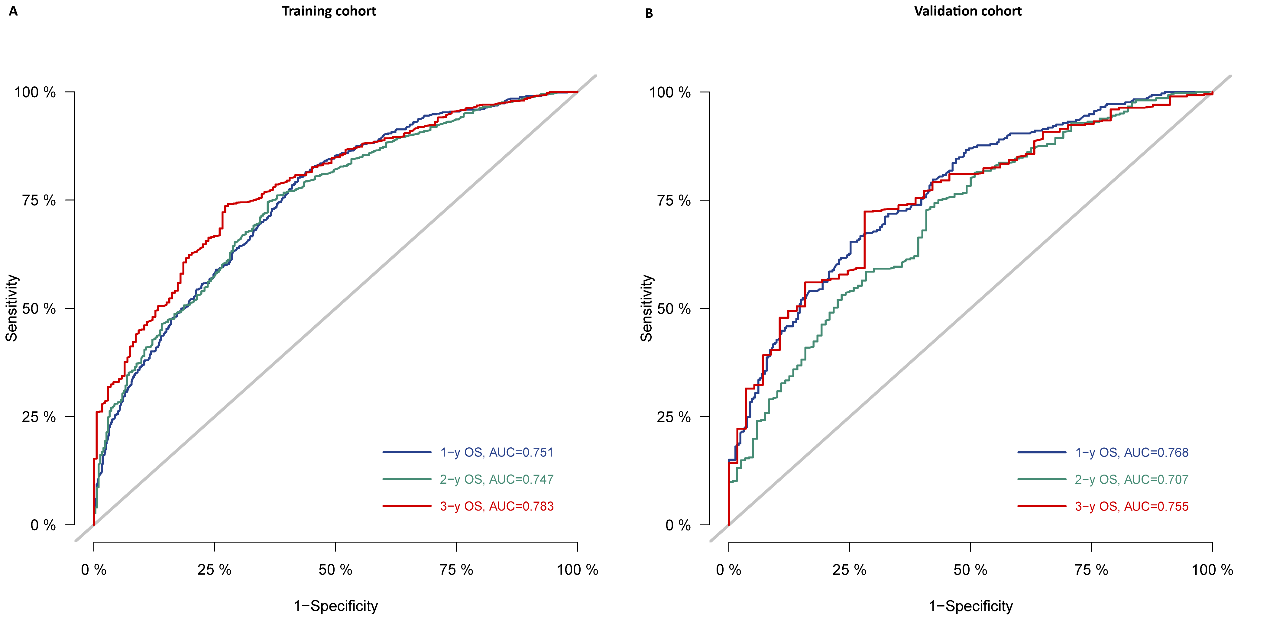
**

**Supplementary Fig 3. Calibration curves of 1-, 2-, and 3-year OS in training and validation cohorts.**

**
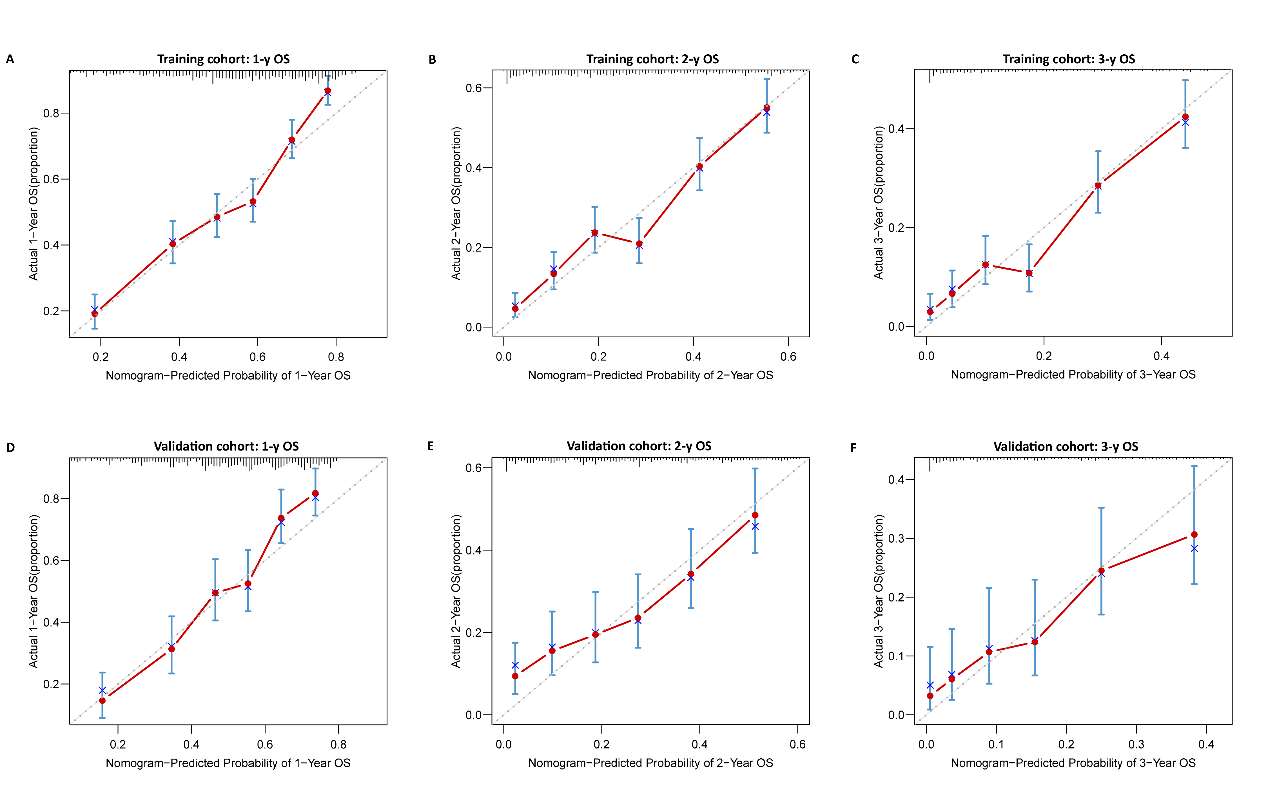
**

A. Training cohort: 1-y OS; B. Training cohort: 2-y OS; C. Training cohort: 3-y OS; D. Validation cohort: 1-y OS; E. Validation cohort: 2-y OS; F. Validation cohort: 3-y OS.
